# Trading Animal Lives: Ten Tricky Issues on the Road to Protecting Commodified Wild Animals

**DOI:** 10.1093/biosci/biab035

**Published:** 2021-04-14

**Authors:** David W Macdonald, Lauren A Harrington, Tom P Moorhouse, Neil D'Cruze

**Affiliations:** Founder and director; Harrington is a senior researcher; Department of Zoology at the University of Oxford, Tubney, Oxfordshire, United Kingdom; global head of wildlife research, World Animal Protection, London, England

**Keywords:** conservation, wildlife trade, animal welfare, demand redirection, sustainable resource use

## Abstract

Wildlife commodification can generate benefits for biodiversity conservation, but it also has negative impacts; overexploitation of wildlife is currently one of the biggest drivers of vertebrate extinction risk. In the present article, we highlight 10 issues that in our experience impede sustainable and humane wildlife trade. Given humanity's increasing demands on the natural world we question whether many aspects of wildlife trade can be compatible with appropriate standards for biodiversity conservation and animal welfare, and suggest that too many elements of wildlife trade as it currently stands are not sustainable for wildlife or for the livelihoods that it supports. We suggest that the onus should be on traders to demonstrate that wildlife use is sustainable, humane, and safe (with respect to disease and invasion risk), rather than on conservationists to demonstrate it is not, that there is a need for a broad acceptance of responsibility and, ultimately, widespread behavior change. We urge conservationists, practitioners, and others to take bold, progressive steps to reach consensus and action.

The noun commodity describes something that can be bought and sold, and as its top illustration of the usage of the verb commodify, the Google dictionary gives “some conservationists have criticized the approach as commodifying nature.” People have commodified nature for centuries, in many and diverse ways: for food; for traditional medicine; for furs and skins, pets, ornaments, collectables, or spiritual artifacts; for their own use or for commercial gain (Roth and Merz 1997). Commercial uses include the rights to hunt, fish, touch, photograph, or watch an animal, as well as to kill or collect it (e.g., Valentine and Birtles 2003, Loveridge et al. 2007). Some forms of wildlife commodification may have beneficial impacts on biodiversity conservation and animal welfare, whereas others may have inimical (i.e., harmful) impacts (Engler and Parry-Jones 2007, Roe 2008, Cooney et al. 2015). Put simply, arguments for benefits from commodification amount to what pays stays (cf. Eltringham 1994), exemplified by natural capital economics (Helm 2015), ecotourism, or community based natural resource management (e.g., Taylor 2009). Indeed, the Convention on Biological Diversity, specifically, anticipates conservation gains from demand for sustainably sourced wild products (e.g., CBD/COP/Dec/14/7; Hutton and Leader-Williams 2003). Arguments that commodification is inimical highlight that overexploitation (the harvesting of species from the wild at rates that cannot be compensated for by reproduction or regrowth) is one of the biggest drivers of vertebrate extinction risk globally (Maxwell et al. 2016) and has been ranked as the second most important driver of change in nature (IPBES 2019).Billions of dollars are exchanged annually on illegal wildlife trade (‘t Sas-Rolfes et al. 2019); the exact value is unknown, but it is overshadowed by the legal wildlife trade, estimated at over US$300 billion per annum (including timber and fisheries; Engler 2008). That the wildlife trade is enormous suggests good potential for beneficial markets (on the basis of what pays stays, and assuming property rights, which govern who can access, use, and benefit from resource use and therefore provide an incentive for long-term sustainable use, are properly assigned; Cooney et al. 2015). But it also masks the details specific to habitats, species, and individuals, which include empty forests (Nasi et al. 2011), endangered species (Maxwell et al. 2016), and poorly treated animals (Baker et al. 2013). These issues are not limited to the illegal wildlife trade, and although some (involving, for example, rhinos, elephants, and pangolins) rightly receive considerable media attention (e.g., Harrington et al. 2018), many others (e.g., those involving smaller vertebrates, and plants, and invertebrates; e.g., Fukushima et al. 2020) don't.

One way or another, wildlife is considered an asset that can deliver ecosystem services, provide food for subsistence, or act as an economically valuable resource or entertainment for tourists. In all cases, where nature is considered as an asset, this is associated with demand for it. Sometimes that demand (although it is not demand itself that is the problem) will, from the viewpoint of animal conservation (and animal welfare; Paquet and Darimont 2010), be inimical. But sometimes it will be a lifeline from extinction.

## Nature's marketplace

Recognizing nature as a key contributor to nations’ wealth, in the 1990s cutting edge conservation hoped to express the value of wildlife through monetizing it (McNeely et al. 1990), whether directly or through incentive measures. Of course, money is not everything and at least some nonhuman animals—and, potentially, even some elements of ecosystems—have intrinsic value that needs to be accounted for in society's conservation audit (Vucetich et al. 2018). The trade in wild animals raises a wealth of challenging considerations, which are bound up in the question of whether elements of nature should be treated as monetized units traded for utilitarian ends or in their own right as stakeholders in the outcome. The trade may be legal or illegal, small or large scale, national or international, consumptive or nonconsumptive; it may have implications, good, bad, or neutral, for animal conservation and animal welfare, and ultimately, it may be sustainable or unsustainable (all of which may be fluid through time and space).

Legal and illegal trade in wild animals involves diverse supply chains, from subsistence hunters lacking alternative sources of income to highly organized international corporations that distribute and market a diversity of wildlife products (Dutton et al. 2013, Phelps et al. 2016). Trade takes place at local markets (e.g., Fa et al. 2006), in farms that produce crocodiles for their skins and pythons and hornbills for the exotic pet trade (Tensen 2016), and increasingly online (e.g., Yu and Jia 2015, Alfino and Roberts 2018, Ye et al. 2020). And the calculated economic value of this commodified nature depends on what is included. Costanza and colleagues (1997) estimated that ecosystem services (including abstract concepts such as the value of carbon, nitrogen, and water cycles) saved humanity bills of US$33 trillion per year. Less abstract commodities also generate sizeable markets; wildlife tourist attractions, for example, are viewed by upward of 6 million paying customers annually (Moorhouse et al. 2015), and wild meat provides a source of protein for 150 million rural households across the global south (Nielsen et al. 2017). In the United Kingdom alone, 40 million pheasants are released into the countryside annually to supply game for shooting (Feber et al. 2020), and this is just one tiny element of the global hunting market (e.g., Loveridge et al. 2007). A single large seizure of pangolin scales may be worth up to US$38 million (Reuters 2019), and the value of rhino horn (in 2013) exceeded that of gold or cocaine (Dickman et al. 2020). As far as we are aware, nobody has attempted to calculate the financial value of all these markets combined, but if they did the number would likely be so large as to defy comprehension.

Two contrasting approaches to reducing inimical aspects of trade can be described in their simplest form as either reducing unsustainable offtakes that supply markets (supply reduction) or reducing the demand for that supply (demand reduction; McNamara et al. 2016, ‘t Sas-Rolfes et al. 2019). Both involve the law and its enforcement, as do many aspects of conservation (Macdonald 2019, Shao et al. 2021), and both involve practical difficulties and ethical dilemmas. Supply reduction (e.g., by enforcement of laws protecting animals from hunting) may involve punishing people who are relatively poor. At the extreme this involves shoot-to-kill policies toward suspected poachers (Duffy 2017, Dickman et al. 2020). On the other hand, punishments that are too lenient will not deter (e.g., Risdianto et al. 2016). For many reasons, supply reduction is difficult when demand is seemingly infinite, and finite wildlife resources subject to such demand require immediate protection. Many wildlife practitioners therefore believe the answer to lie in demand reduction (or other demand-side interventions; e.g., Veríssimo et al. 2012). But reducing demand will, at its root, require behavior change (Wallen and Daut 2018) on a vast, perhaps unprecedented scale.

Taking a wider perspective, wildlife protection efforts hope to reduce the inimical impacts on conservation and animal welfare that arise from demand for wildlife, while simultaneously hoping that demand for wildlife can permit wildlife to pay for its own protection. These contrasting aspirations are both inspired by a desire to conserve wildlife: reducing demand for (and supply of) many aspects of traded nature because they are damaging (or seem morally inappropriate) for conservation and animal welfare, while increasing demand for (and supply of) other aspects of traded nature, because in some currency (including money) this gives nature value on which its survival may depend.

Overall, given the continuing decline of biodiversity (IPBES 2019), the prominence of overexploitation as a driver of that decline (Maxwell et al. 2016, IPBES 2019), and the questionable treatment of many animals in trade (Baker et al. 2013), the global trade in wildlife contains too many cases that currently appear to be more inimical than beneficial to biodiversity, conservation, and welfare (the quest for remedies to this observation should be alert to unintended consequences—see Conclusion).

In the present article, drawing insights from the direct experience of our own research unit (the Wildlife Conservation Research Unit at Oxford University), over more than 30 years (and with the two contrasting aspirations outlined above in mind), we ask whether the commodification and the subsequent trade of nature is able to deliver positive conservation outcomes under current circumstances. In doing this, we highlight 10 tricky issues (these are not necessarily mutually exclusive, and others may identify more or fewer) with the aim of helping to understand the impediments to wildlife trade management approaches and implementation. The intended purpose of our essay is to stimulate debate around the issues that we raise, in the hope of identifying a path toward consensus on how best to weigh the balance in favor of wildlife. The issues themselves are not, for anyone familiar with wildlife trade, necessarily particularly shocking, controversial, or unknown. The controversy, instead, arises in their potential solutions, and, for that reason alone, we highlight these issues in the present article in the hope not of identifying unique or novel problems but of stimulating discussion as to how they might be overcome—before it is too late.

## Issue 1: It's not only illegal wild animal trade that's problematic

Legal wildlife trade is enormous, and its legality—whether carried out under CITES (the Convention on International Trade in Endangered Species of Wild Fauna and Flora) or another regulatory framework—does not necessarily imply sustainability, sufficient protection of animals’ welfare, or adequate safeguarding of natural environments or human health.

Between 2012 and 2016, the CITES Trade Database (http://trade.cites.org) recorded the export of 11 million individual live (wild sourced, ranched, and captive bred or born) wild vertebrates (2.2 million per year on average), of 1316 different species, exported from 189 different countries (Can et al. 2019), most of which were likely destined for the exotic pet trade. Most were en route to the United States and the European Union (Can et al. 2019; see also Bush et al. 2014, Harrington 2015). A similar number of dead wild animals are legally traded across the globe as parts or as wildlife products; the data in Harfoot and colleagues (2018) recorded a total of 4.5 million whole animal equivalents of (live and dead) vertebrates exported in 2014 alone. Many more individuals and species are traded without CITES regulation (which concerns only those species considered to be threatened, or at risk of becoming threatened, in the wild by trade, and traded internationally); consider domestic consumption of bushmeat (see Issue 8), commercial fisheries (which captured 84.4 million tons of fish from the seas in 2018, FAO 2020), and those species that are traded internationally but not listed on CITES appendices (the private online commercial trade of live reptiles alone involves more than 2000 species in unknown numbers, three-quarters of which are not CITES listed; Marshall et al. 2020; see also Auliya et al. 2016). Combining information from CITES trade records and the IUCN Red List (www.iucnredlist.org) suggests that more than 7000 (approximately 24% of all) extant vertebrate species are involved in wildlife trade (Scheffers et al. 2019; an underestimate for some taxa, e.g., reptiles; Marshall et al. 2020), double this number if fish are included, plus (a presumably hugely underestimated) several thousand invertebrate species (Fukushima et al. 2020).

In most cases, the conservation consequences of these enormous legal trades are unknown because of insufficient monitoring of the trade and of the involved species’ wild populations. In the reptile and amphibian trade, for example, the import and export of millions of individuals into and out of the United States is recorded every year, but the species to which they belong is often not, which, coupled with the absence of baseline biological data, means that sustainability is impossible to assess or ensure (Schlaepfer et al. 2005; see also Auliya et al. 2016, Harris et al. 2017). Similarly, over 50 wild carnivore species are trapped for their skins, generating 3.5 million pelts in the United States in 2014 (under US state, federal, and tribal agency management through the Association of Fish and Wildlife Agencies, www.fishwidlife.org), many without population monitoring or adequate regulatory protection (Harrington et al. 2017 and the references within it). Even for CITES-regulated trade, sufficient data are lacking to demonstrate nondetriment (a key requirement of CITES, articles III and IV, www.cites.org) in many cases (e.g., Smith et al. 2011, Natusch et al. 2019). For fur bearers, not all government-endorsed trapping methods are considered humane by animal welfare scientists (e.g., Iossa et al. 2007), and for species traded live, there are welfare impacts for the individuals involved at all stages of the trade chain (Baker et al. 2013, Ashley et al. 2014, Bergin and Nijman 2019, D'Cruze et al. 2020b).

Global trade in live animals is also a major source of invasive species (Hulme 2009, Lockwood et al. 2019). The substantial feral turtle populations in the Yangtze River Basin are just one example of the broader consequences of a thriving trade in nonnative reptile pets, hundreds of thousands of which are sold online within China via China's Taobao.com e-commerce site every month (Liu et al. 2020). In addition, national and international animal movements create a significant and growing pathway of risk for the spread and release of zoonotic disease (Karesh et al. 2005, Travis et al. 2011 and the references within it). Wild animals are thought to be the source of at least 70% of all emerging infections (Taylor et al. 2001) and data from the World Organisation for Animal Health contained, between 2008 and 2016, an inexhaustive list of at least 82 zoonotic pathogens of mammals, amphibians, birds, and reptiles causing 3131 disease cases in 54 countries as reviewed by Can and colleagues (2019), with tragic prescience regarding the COVID-19 virus (Zhou et al. 2020). Currently, there is little or no disease screening of wild animal imports (Kolby 2020).

## Issue 2: Existing wildlife trade legislation contains holes

Although there are national and international laws and agreements in place to ensure that trade in live wild animal species and their body parts is sustainable, humane, and safe, complexity and, in many cases, inconsistency too often render existing regulations ineffective.

CITES, as one of the principal regulatory frameworks for international trade in animals and plants, is limited by an incomplete species list, an underlying lack of knowledge of listed species (i.e., population status, offtake levels and sustainable harvest rates, see Issue 1) and party compliance (Phelps et al. 2010). In addition, records held in the database are often inaccurate, incomplete (e.g., taxa identified only to class), and inconsistent (e.g., units, and traded volumes reported by exporter and importer differ, and source codes are not mutually exclusive), meaning that data can be easily misinterpreted and misreported (e.g., Phelps et al. 2010, Robinson and Sinovas 2018, and the references within them). Assessing the extent of illegal trade (and irregular trade, in which permissions and paperwork are lacking) is, by virtue of its clandestine nature, difficult (‘t Sas-Rolfes et al. 2019). However, beyond CITES regulations and the difficulties of quantifying illegal wildlife trade, sometimes the answer to the most basic question—what is legal and what is illegal—is not straightforward.

The Indian star tortoise (Geochelone elegans), for example, is a popular exotic pet but within its range states of India, Sri Lanka, and Pakistan, national laws make it illegal to privately possess or commercially trade in wild caught individuals, and no large-scale commercial captive production facilities are known to exist (D'Cruze et al. 2015). However, until its transfer to CITES appendix I at the 2019 Conference of the Parties meeting (CoP18, www.cites.org), commercial trade in Indian star tortoises from these range states could still take place internationally (and thereafter domestically in nonrange states) under CITES appendix II permit. In Thailand, for example, the Indian star tortoise was among the most commonly observed chelonians at the infamous Chatuchak market (Chng 2014). According to CITES records, Indian star tortoises in Thailand were, historically, imported from Kazakhstan (a nonrange state) but there are no import records into Kazakhstan to demonstrate procurement of captive breeding stock. Between 2008 and 2013, Thai enforcement authorities seized almost 6000 individuals (in 15 cases; Chng 2014), but the difficulties in distinguishing wild caught and captive bred individuals (D'Cruze et al. 2015) meant that identifying those that were illegally traded was almost impossible. Prior to CoP18, the same situation prevailed for pet small-clawed otters (Aonyx cinereus) in Japan (where, as a nonnative species previously listed on CITES appendix II, now appendix I, they were not protected and could be legally kept and sold by individuals, pet cafes, and breeding centers) but were likely sourced from range states in Southeast Asia (where hunting, keeping or breeding of this species is illegal; see Harrington et al. 2019 and the references within it). For both otters and tortoises (just two examples), weaker or nonexistent domestic legislation in nonrange states provided legal loopholes. Indeed, ‘t Sas-Rolfes and colleagues (2019) suggested that trade-related activities (from harvest to end use) are seldom uniformly illegal along the entire trade chain, creating complexities throughout.

Complexities in trade regulation also arise from taxonomic uncertainties and changes (Macdonald 2019). In China, newly discovered phylogenetic relationships and outdated protected species lists mean that the names of several threatened species are inconsistent with national legislation (Zhou et al. 2016a, Gong et al. 2020). Leaf monkeys (traded as pets and bushmeat; Funk 2016) are included on the List of Fauna under Special State Protection (LFSSP) 1989 as Presbytis spp. But former Presbytis spp. are now designated as belonging to one of three genera: Presbytis, Trachypithecus, and Semnopithecus. Presbytis only occur outside China, which leaves Chinese species of leaf monkey (all of which are Trachypithecus or Semnopithecus spp.) unprotected by national laws. Similarly, traders in Burmese pythons (Python bivittatus) in China evade prosecution under national legislation designed to protect both native species (under the LFSSP) and nonnative CITES-listed species (under the 1993 notice by the former State Forestry Administration; see Gong et al. 2020) because Python bivittatus (formerly considered a subspecies of the Indian python, Python molurus) is not recognized as a native species by Chinese lawmakers and is therefore not named on the LFSSP, but it is (correctly) listed as native to China by CITES, and so is also not protected under the 1993 notice. They also evade prosecution under CITES because (as a native species) specimens confiscated in China are only in violation of CITES if they can be proved to have been trafficked across China's borders. These issues are not restricted to China: Of the 17 megadiverse countries, only 5 have updated their protected species lists since 2007 (Zhou et al. 2016a). At an international level, although CITES policy is to amend Appendices in such cases, delays in the process hinders ongoing conservation efforts (Zeng et al. 2019).

Deliberate misnaming can also be used by traders to similar effect, using code words (Alfino and Roberts 2018) or deceptive labeling. The dried hemipenes of protected Varanus lizards (for which trade is illegal), for example, are sold by online retailers as Hatha jodi, the roots of a Himalayan plant purported to have curative powers to which varanid hemipenes show a remarkable resemblance (Bhattacharya and Koch 2018, D'Cruze et al. 2018a, Rajpoot et al. 2018).

With respect to animal welfare, although the Agreement on International Humane Trapping Standards (between the European Commission, Russia, Canada and the United States, Council Decisions 98/142/EC and 98/487/EC) attempts to ensure humane trapping methods for all skins imported into the European Commission (where several major auction houses operate), not all furbearers (notably, American mink, Neovison vison, hundreds of thousands of which are trapped annually) are included (Harrington et al. 2017, Proulx et al. 2020).

## Issue 3: Nations are insufficiently prepared to deal with confiscations

Intercepting contraband is surely a good thing and may diminish the profitability of illegal wildlife trade, but, where wildlife trade involves live animals, the treatment of rescued animals is also crucial. Poor planning or a lack of preparedness results in potentially ineffective conservation interventions that may undermine animal welfare goals, amid little reporting or accountability.

Startling numbers of animals traded illegally or without the required paperwork are impounded. In just one Chinese province, Yunnan, over 12,000 native live reptiles were seized between 2010 and 2015, of which Zhou and colleagues (2016b) estimated fewer than 30% were returned to the wild; because the euthanasia of rescued wildlife is not permitted in China, the rest went to sanctuaries (reputedly crowded and underresourced) or even into the pet-food trade. Of 155 native birds among the seizures, all were released back into the wild but into unfamiliar release sites and without follow-up monitoring (Zhou et al. 2016b). Only 98 of the 326 pangolins placed in the Yunnan Wildlife Sanctuary between 2008 and 2014 survived the transfer (Zhou et al. 2014) and similarly low survival rates have been reported for rescued slow lorises Nycticebus spp. in wildlife centers (Fuller et al. 2018). Presumably because of logistical challenges, none of five endangered Asiatic elephants (Elephas maximus) rescued in 2005 have been returned to Myanmar (Zhou et al. 2016b).

Scale the Yunnan problem globally, and the numbers of detainees, and (in the absence of monitoring) ignorance of their fates, become huge. Between 2010 and 2014, a subset of confiscated and seized shipments reported to CITES (those reportedly being legally reexported; in some cases, being returned to export countries, and presumably only a tiny fraction of all seizures of animals at borders) included 64,143 individual live animals, of 359 species, 19% of which were threatened (and therefore of conservation concern; D'Cruze and Macdonald 2016) and all of which were vulnerable and displaced from their natural range and habitats. Using a larger (but also incomplete) database of global seizures, UNODC (2016) referred to seizures of more than 4000 different species of mammal and reptile between 1999 and 2015 (approximately 60% of the total animal and plant species reported). The relevant management authorities are expected to deal with confiscated animals in a humane manner that maximizes their conservation value and does not promote further unsustainable illegal trade. Acceptable options include maintaining them in captivity, return to the wild or euthanasia (CITES Resolution Conf. 17.6), each of which has different ramifications for animal welfare and conservation and, often, considerable logistic challenges. However, information on the disposal of seized animals is sparse, and, even under new CITES illegal trade reporting requirements (CITES Resolution Conf. 11.17 (Rev. CoP17)) that came into effect in September 2018, the inclusion of such details in illegal trade reports are desirable rather than obligatory (CITES Notification 2018/009). Indeed, although the reports themselves are mandatory, by mid-2019, only 59 of 183 (32%) countries had submitted a report (Lopes et al. 2019; and the contents of these reports are not publicly available).

Surrender of illegally kept animals in response to awareness raising campaigns faces the same issues: Rescue centers are full (e.g., Karokaro et al. 2017), but liberation risks low survival for animals kept as pets, habituated to humans, that no longer have the skills for survival in the wild (e.g., Waples and Stagoll 1997) and also risks the potential spread of disease (Gray et al. 2017).

## Issue 4: Bans may have unintended consequences for other species unless they are extended to possible substitutes

Making a particular wildlife trade illegal is unlikely to deliver benefits in the absence of effective enforcement and in the presence of unabated consumer demand, which can not only lead those involved in illegal trade (knowingly and unknowingly) to continue and even expand criminal operations if consumers remain willing to pay a price that outweighs potential penalties but can also drive alternative trades, with unintended consequences for the conservation status and welfare of analogue species that are not afforded the same protection.

The effectiveness (and appropriateness) of targeted trade bans (such as that resulting from a CITES appendix I listing of a species) is a hotly debated issue (e.g., Rivalan et al. 2007, Conrad 2012, Challender and MacMillan 2014a, 2014b, Phelps et al. 2014, Challender et al. 2015, Weber et al. 2015, Bonwitt et al. 2018), but beyond the impacts (positive or negative) on the target species, there can also be (unforeseen) negative impacts on other species that are not afforded the same protection. As the availability of Asian pangolin species declined (in this case, because of overhunting and population decline, rather than a ban on hunting, but the effect is the same), African pangolin species were increasingly sourced to supply pangolin scales for Chinese traditional medicine (Challender and Hywood 2012, Ingram et al. 2019). In the present article, we focus on tigers and lions, for which failure to address consumer demand has meant that partial legislation and actions to protect one species of big cat in Asia (tigers) gave rise to unintended impacts on others (lions) in Africa and potentially beyond (jaguars in South America).

Celebration of the 1987 ban on international tiger (Panthera tigris) trade (including, in 2007, prohibition of commercial captive breeding of tigers for trade in their parts or derivatives; CITES decision 14.69) will for some have been marred by the facts that the market has simply turned to substitutes and that over 5000 tigers are still typically held in suboptimal welfare conditions across 200 facilities in China (EIA 2013). In 2005 African lion (Panthera leo) DNA was detected in “tiger” bone strengthening wine, and in three incidents in 2007 Asiatic lions (Panthera leo persica) were poached for their bones in India's Gir National Park (Williams et al. 2015, and the references within it). In 2015 Williams and colleagues (2015) presented trade data suggesting a developing market, with CITES permits issued to lion breeders in South Africa to export captive-bred skeletons to Asia at a rate that increased tenfold between 2008 and 2011. This was accompanied by an increase in captive-bred (“canned”) lion hunting by Asian clients in South Africa and in the export of lion “trophies” by Asian customers (Williams et al. 2015). Apparent inconsistencies between the nationality of foreign hunters and the export destinations of their trophies raised suspicions that some hunts were motivated to supply the wildlife trade with lion body parts (although the hunts were entirely legal, the subsequent commercial trade in lion bones was not). At the 2016 CITES Conference of the Parties (CoP17), measures were adopted requiring a quota on South Africa for bones, bone pieces, bone products, claws, skeletons, skulls, and teeth sourced from its captive lions and traded for commercial purposes, alongside a zero quota for lion parts and derivatives (but not skins), of wild origin elsewhere (Bauer et al. 2018). This quota is notably inconsistent with CITES decisions (Dec. 14.69) on tiger farming that specifically prohibit breeding for commercial purposes, given the apparent intermingling between the two trades, and was presumably influenced by political and situational aspects of the negotiation process (Bauer et al. 2018).

The impact of this trade on the conservation status of wild lion populations is largely unknown and thus far there is little evidence of increased poaching of wild lions to supply the trade (Williams et al. 2017, Coals et al. 2020a). In South America, however, recent increases in seizures of jaguar (Panther onca) body parts (predominantly canine teeth) with links to China, has raised concerns regarding potential impacts on the status of wild populations and long-term conservation efforts for jaguars there, although it is not currently clear whether the parts seized are intended for the medicinal bone trade or for jewelry or amulets, and whether they are intended as market complements or substitutes for tigers and other big cats (Morcatty et al. 2020).

Globalization increases opportunities for product substitution and facilitates such shifts onto analogue species (Esmail et al. 2020), which may occur in different countries, on different continents, and can be unpredictable.

## Issue 5: Wildlife farming does not always help

Insofar as demand continues to outstrip the potential of wild populations to supply an obvious and often voiced solution lies in commercial captive breeding. But some animals are difficult to breed in captivity, stocks may be dependent on wild sources (with potential impacts on wild populations), animal welfare conditions are often poor, and consumers may, in any case, prefer wild-sourced products (which risks farmed products establishing a market and increasing demand for wild counterparts).

Over recent decades CITES trade has shifted significantly from wild-sourced to captive-sourced wildlife products across most taxa (Harfoot et al. 2018, see also Robinson et al. 2015). Possible reasons for the shift include reliability, quality assurance, public perception regarding exploitation of wild animals, CITES controls on wild harvest, or declining availability of wild animals (Harfoot et al. 2018). For crocodilians, ranching and farming has generated significant conservation gains including suppression of poaching, and population recovery following overexploitation for their skins in previous decades (see e.g., spectacled caiman, Caiman crocodilus; Sinovas et al. 2017, and see Dutton et al. 2013, Moyle 2013, and the references within them). However, some species are difficult, sometimes impossible, to keep or to breed in artificial or confined environments. Pangolins, for example, have an extremely specialized diet and there have been few records of successful captive reproduction (Hua et al. 2015); for this species, farming is very unlikely to displace wild collection in the near future (Challender et al. 2019).

A captive-bred supply can also complicate the policing of illegal supply lines and offer opportunities for laundering (Fischer 2004), and Harfoot and colleagues (2018) suggested that the observed increase in captive-sourced wildlife in trade may be due, at least in part, to misreporting. It is unlikely, for example, that Chinese breeding centers can produce the tens of thousands of crab-eating macaques exported to the United States for the biomedical and biodefense market without wild-caught supplementation (Eudey 2008) and similar concerns have been raised regarding commercial breeding of Tokay geckos (Gekko gecko) in Indonesia for the pet trade (Nijman and Shepherd 2015, see also Lyons and Natusch 2011, Janssen and Chng 2018). Tensen (2016) referred to several captive bred species in trade (including civets, cane rats, porcupines, green pythons, Siamese crocodiles, and Papuan hornbills) dependent (or likely dependent) on wild stock, and a detailed analysis of ball python ranching operations in Togo suggest that current practice is unsustainable at best and likely linked with perceived nationwide declines in the species (D'Cruze et al. 2020a).

For species that can be bred in captivity, without dependence on wild sources, minimum standards for animal welfare are not always met. Crocodile farms have received criticism in the media for poor animal conditions (e.g., Keletso 2015), and the small wire cages that house palm civets (Paradoxurus hermaphroditus) to excrete coffee beans for tourists (Carder et al. 2016) are questionable at best. Green turtles (Chelonia mydas) bred in the Cayman Islands to produce a regional delicacy for tourists suffer high mortality rates (D'Cruze et al. 2014) and physical injury and disease and abnormal behaviors associated with overcrowding and captivity-related stress (Arena et al. 2014).

Is the customer content with the substitute? Gratwicke and colleagues (2008) reported that 71% of consumers of tiger-based products preferred ingredients from wild tigers in traditional medicine, and Chinese consumers of bear bile indicated that they were willing to pay considerably more for wild-sourced than farmed ingredients (Dutton et al. 2011). Preferences, however, are complex (and do not necessarily reflect behavior, which is also influenced by legality, price, and availability; Hinsley and ‘t Sas-Rolfes 2020), and these attitudes may be changing as consumers become more conservation conscious (Moorhouse et al. 2020). But it is nevertheless noteworthy that despite increasing trade in lion bones (as an apparent substitute for tiger bones; issue 4), in China and Vietnam, tiger bone wine is greatly preferred over lion bone wine (Coals et al. 2020b). Exotic pet buyers may prefer captive-bred sources (e.g., Harfoot et al. 2018) but that is not to say that they are aware (or correctly informed) of the provenance of their purchases (between 2007 and 2016, 11,000 of the 305,000 ball pythons imported by the European Union for the exotic pet trade were sourced directly from the wild in their West African range states; Harrington et al. 2020).

Assessing the circumstances under which wildlife farming may be beneficial to species conservation is complex (Moyle 2013) but can be broadly summarized as the need to fulfill four criteria: to provide a true substitute for the wild product, to meet demand and not prompt a significant increase in unsustainable demand for the wild product, to be more cost efficient than the hunting or harvest of wild individuals, and to not rely on wild animals for restocking and demonstrably disallow laundering (see Biggs et al. 2013). A review of the existing scientific literature by Tensen (2016) suggested that only a minority of species is likely to meet these criteria. If acceptance by a global public is dependent on higher welfare standards the number will likely be even fewer.

## Issue 6: Demand reduction has not yet fulfilled its promise

Given the issues associated with targeted trade bans and wildlife farming, a key hope is to reduce consumer demand for unsustainably sourced wild products through educational and public awareness campaigns drawing awareness to consumer responsibility. However, the difficulty of influencing the complex of attitudes and behaviors that drive human consumption means that demand reduction interventions have not yet fulfilled their logical promise.

There have been multiple calls for demand reduction in both the academic and grey literature (Veríssimo et al. 2012, Challender et al. 2014c, Burgess 2016). Few demand reduction initiatives, however, are currently underpinned by an adequate understanding of target audiences’ motivations (Thomas-Walters et al. 2020a), and there is a general lack of approaches to understanding more nuanced attitudinal data, such as how price might influence purchase choices, which consumers are most likely to change their behavior, and what might trigger them to do so (Burgess et al. 2018).

Illustrating the necessity of getting the message right, Moorhouse and colleagues (2017a) tested the influence of different types of information on the desires of 1305 would-be owners of wildlife pets. The participants stated desire to own a given pet was not affected by detriment to the pet's welfare nor to its conservation, but the respondents were put off by the risks of legal action or disease. In this case, the type of message made a difference; self-interest appeared a more potent influencer of this group than animal conservation or welfare.

In contrast, 1600 consumers of traditional Chinese medicine (TCM) appeared not to be influenced by the content of information intended to reduce demand by appealing to consumers’ concerns over conservation or welfare impacts, or about personal harm, and this was particularly the case for regular, compared with occasional, TCM buyers (Moorhouse et al. 2020). Regular buyers, however, were the most likely to state a willingness to purchase alternative products made from herbal sources, and so Moorhouse and colleagues (2020) concluded that a more effective way to reduce the trade in animal origin medicines might be to attempt to redirect demand onto potentially less damaging products, rather than reduce demand through information campaigns.

Identifying who should deliver the message and through which channels is also key (Davis et al. 2020). However, behavior change remains difficult to achieve and outcomes may be unpredictable (Thomas-Walters et al. 2020b). Conservation marketing has huge potential for redirecting or reducing demand for wildlife products (Wright et al. 2015, Wallen and Daut 2018, Veríssimo 2019), but there is currently little evaluation of impact or follow-up (Veríssimo and Wan 2019; see also ‘t Sas-Rolfes et al. 2019). Demand reduction (through whatever mechanism) is unlikely to provide quick and easy solutions (Thomas-Walters et al. 2020b).

## Issue 7: For win–win outcomes, market regulation is unavoidable

Directing consumption onto wildlife usages that are not only less damaging but that are demonstrably sustainable theoretically creates a win–win for consumers and wildlife: Consumer desire is satisfied and wildlife can pay for its own protection. As an example, wildlife tourist attractions (WTAs), and wildlife tourism in general, provide a commercial nonconsumptive wildlife trade, whereby consumers pay to see wildlife rather than to consume or own it, and therefore have the potential to provide such win–win outcomes. Currently, however, wildlife tourists’ impacts on wildlife are often detrimental, and, in the absence of regulation, it is likely the ill effects will outbalance any positive impacts of the tourism market for species conservation.

Tourism is a major global economic driver, accounting for 7% of total global exports and providing 1 in every 10 jobs worldwide (United Nations World Tourism Organization, UNWTO, tourism data dashboard, www.unwto.org). In 2018, global tourism was worth US$1.7 trillion, increasing at about 5% per year, and there were 1.4 billion international tourist arrivals (UNWTO tourism data dashboard). Wildlife tourism has long been the leading foreign exchange earner in several countries (Reynolds and Braithwaite 2001) and a substantial proportion of this trillion-dollar industry. Involving everything from photo and hunting safaris to zoos, one substantial and growing category—WTAs (Moorhouse et al. 2015, 2017b)—offers tourists opportunities to interact with nondomestic animals, in captivity or the wild. WTAs include touching, feeding, and taking selfies with tigers, lions, and dolphins in captivity; trekking to observe gorillas and gibbons in the wild; visiting bear bile, sea turtle, and civet coffee farms; viewing rehabilitated or rescued animals (e.g., orangutan sanctuaries); and watching wildlife-based shows (e.g., snake charming or dancing macaques).

A huge diversity of species is involved, which in Latin America is disproportionately represented by mammals, and specifically those mammals classified as Vulnerable on the IUCN Red List of Threatened Species (perhaps because they are rare enough to be considered exciting but not so rare that they could not be reliably found; D'Cruze et al. 2018b). Moorhouse and colleagues (2015) estimated that globally 24 types of WTAs (half of the 48 types they considered) negatively affected the welfare status of 230,000–550,000 individual animals and were inimical to the conservation status of the species of 120,000–340,000, whereas only six WTA types (affecting 1500–13,000 animals) had net positive impacts on both species’ conservation and individual animals’ welfare. Broadly defined as nonconsumptive, many of these WTAs involved animals captured from the wild and held in captivity (restrained on chains or leashes, or held in cages in between tourist visits; e.g., D'Cruze et al. 2017), lost to conservation as surely as if they had been eaten or hunted. There may or may not be a payback in tourist education (e.g., Ballantyne et al. 2011, Fernández-Llamazares et al. 2020), but balanced against this is also any potential cost to the welfare of those individuals (e.g., Constantine et al. 2004, Alves et al. 2011, Ranaweerage et al. 2015, Schmidt-Burbach et al. 2015, D'Cruze et al. 2017). Tourism involving wild animals can foster the protection of their habitats (e.g., Herzl 2019) but can also be damaging to the conservation of species and distressing for individuals (Belicia and Islam 2018). Overall, the 24 types of WTA assessed by Moorhouse and colleagues (2015) hosted an estimated 3.6–6 million tourists per annum, of whom about 60% were likely to have supported, through patronage and whether knowingly or not, attractions with negative impacts.

WTAs are not globally regulated, and most travel trade associations ignore their responsibilities toward animal welfare (Font et al. 2019), so in many countries their standards are determined by customer patronage (Moorhouse et al. 2017b). But wildlife tourists do not always consume responsibly. A global survey revealed participants to discern and prefer beneficial WTAs but only if first primed to consider welfare and conservation (Moorhouse et al. 2017c).

## Issue 8: Extractive sustainable use may increasingly be unrealistic

There are startlingly large numbers of animals in various fluxes of commodification and the vision of extractive sustainable use is rapidly becoming a mirage. Human population sizes and rates of usage have risen so quickly, and the number of species and habitats in which consumption could be sustainable has dwindled so rapidly that the concept surely begs for reevaluation.

Commodification's history goes back millennia so one might ask if people have used wildlife for so long, what's the problem with continuing to do so? Many twenty-first century conservation biologists were educated in a mindset that promoted sustainable use as a route to the protection of wildlife and the development of people. The hope was that commodifying wildlife (e.g., through tourism, or sustainable rates of consumptive extraction) could allow nature to pay for its own existence and to financially and socially benefit human communities living with wildlife. This hope was recently paramount in the remarks of several southern African heads of state at the aforementioned African Wildlife Economy Summit in 2019, where dissent from this view appeared to be cast as naive or neocolonial (e.g., Garland 2008). Nevertheless, although the United Nations’ 17 Sustainable Development Goals (www.un.org/sustainabledevelopment/sustainable-development-goals), and their fundamental principles (notably poverty alleviation, attention to international law and human rights, and appropriate, sustainable custody and use of nature) are timelessly worthy, the current reality of human use of commodified natural products—driven by the resource use of 7 (soon to be 10) billion people—is of rampaging forest loss (e.g., IPBES 2019); massive losses of large apex predators in marine, terrestrial, and freshwater ecosystems worldwide (e.g., Estes et al. 2011); roads penetrating into every wilderness (e.g., Laurance and Burgués Arrea 2017); and exponentially rising numbers of human users (using increasingly powerful and efficient technology; Felbab-Brown 2011) affecting an exponentially shrinking wildlife resource, subject to an increasing number of compounding pressures. These trends do not undermine the principle of sustainable use, but suggest that the number of contexts (species, places, forms of exploitation) where resources are sufficiently intact to make extractive sustainable use a realistic aspiration is shrinking.

The use of bushmeat illustrates the scale of the problem: From a single region of West Africa (the Cross-Sanaga forests of Nigeria and Cameroon), approximately 800 kilograms of wild meat is extracted for sale per square kilometer of forest every year (Fa et al. 2006), and in this 35,000 square kilometer study area, the annual number of reptiles, birds, and mammals sold by the rural and urban population was close to a million. Across the tropics, an estimated 6 million tons of animals (mostly ungulates and rodents) are extracted every year (Nasi et al. 2011 and the references within it). Extraction at this scale is unsustainable, particularly for large bodied or slowly reproducing species (e.g., Ripple et al. 2019), especially given the shrinking habitat and proliferation of road access. More than a half of the ocean is industrially fished, and a third of marine stocks are harvested at unsustainable levels (IPBES 2019). For thousands of species, smaller scale uses (e.g., as pets) may simply be the last straw for populations already affected by habitat degradation and human encroachment (e.g., Harrington et al. 2019).

## Issue 9: Facilitators fail to recognize or shoulder their responsibilities

If trade can be sustainable, humane and safe, who should be responsible for regulating it? There are numerous and varied points at which consumers interface with wildlife products or experience wildlife trade, both positive and negative. This means that there are numerous and varied ­facilitators—or gatekeepers—of consumption far beyond border controls and the export or import of CITES-listed species. In the absence of sufficiently broad global regulation, and in the face of ill informed consumers, responsibility falls on gatekeepers. But this responsibility is often unrecognized, in some cases even by the gatekeepers themselves.

A relatively new, but major, facilitator of the global wildlife trade is the internet, and the various websites, apps, and networking platforms that it supports. Online marketplaces and social media sites are used increasingly by both legal and illegal traders (e.g., Yu and Jia 2015, Thong et al. 2019, Liu et al. 2020, Siriwat and Nijman 2020); these platforms enable traders efficiently to procure wildlife, to expand their consumer base via extensive interconnected networks (e.g., Lavorgna 2014), and to set up at little cost (Brenner 2002). E-commerce facilities (e.g., Facebook marketplace or China's Taobao.com) are increasingly integrated into these platforms to take advantage of the networking capabilities provided (e.g., Liu et al. 2020), and sales also take place directly over social media (often via extensive networks of interlinked interest groups; e.g., Hinsley et al. 2016). Websites, social media, and encrypted communication apps can be interlinked to provide efficient and secure transactions (e.g., Yu and Jia 2015). Beyond advertising and facilitating sales, social media activity related to purchase or ownership of wildlife products may also drive demand (e.g., Kitson and Nekaris 2017, Harrington et al. 2019, Liu et al. 2020).

Facebook posts by just two wildlife exporters in Togo, West Africa, contained, over 4 years, advertisements for 200 species for sale and export as pets—predominantly reptiles but also birds and mammals (belonging to 7 and 10 different orders, respectively), amphibians, and invertebrates (e.g., spiders, giant millipedes, praying mantis and snails; Harrington et al. 2021). Seven percent of vertebrate species observed on these two accounts were listed on the IUCN Red List as Threatened, 49% had restricted ranges (and were therefore vulnerable to overexploitation), and 34% were of unknown conservation status, although only 22% were listed on CITES appendices and therefore regulated in trade (Harrington et al. 2021). Thirty-five percent of all known reptiles are traded online (Marshall et al. 2020). At least 70% of 259 social media posts featuring trade in endangered wild-sourced grey parrots (Psittacus erithacus and Psittacus timneh) between 2014 and 2017, showed trade that was likely in breach of national laws or CITES regulations, with between 26% and 44% of posts showing transactions apparently not reported by CITES parties (Martin et al. 2018). Images posted on social media reveal inadequate welfare standards, during capture, in holding facilities, and during transport (e.g., Martin et al. 2018, Harrington et al. 2021).

However, online wildlife trade activities are currently not routinely monitored or regulated by hosting platform companies, with actions largely left up to users (Esmail et al. 2020). Even for illegal wildlife products there is little evidence of trade on the dark web, suggesting a lack of policing on the surface web (Harrison et al. 2016). Self-regulatory mechanisms within the industry (e.g., the Global Coalition to End Wildlife Trafficking Online, www.endwildlifetraffickingonline.org) are commendable but often reliant on reports by the public and almost exclusively focused on illegal activities. For live animals, some social media sites prohibit commercial sales (predominantly for animal welfare reasons), but there are no regulatory watchdog institutions (and any proposed watchdog mechanism would likely be problematic in terms of personal data protection and national privacy laws; Esmail et al. 2020).

At a global level and across all wildlife markets, a variety of platforms and bodies host or oversee activities relevant to wildlife trade; for example, in addition to the direct role of CITES, and to draw attention to just a few, China's national medical insurance influences the products used in TCM, the aviation industry facilitates transport (and through the International Air Transport Association's Live Animal Regulations the conditions under which live animals travel), the World Organisation for Animal Health monitors the human health impacts of the pet trade, the World Association of Zoos and Aquariums oversees the actions of zoos and aquaria, and TripAdvisor and Google provide potentially influential reviews of wildlife tourism venues. The problem is that in the absence of any overarching body, and given resource limitations and commercial interests, each defers to the others to address impacts on sustainability, legality, and humane standards.

## Issue 10: A positive list might work better than a negative one

For legislation to facilitate effective enforcement, the penalties such as fines and jail sentences for illegal trade must be greater than the profits. More broadly, for the vast (and predominantly legal) wildlife trade, the overarching principles of regulation and legislation must be sufficiently sensitive to risk and probability in biological systems to safeguard the long-term survival of species ahead of short-term financial gain. But regulatory systems are disjointed, and the burden of proof and the balance of responsibility are typically upside down when it comes to wildlife law.

CITES is just one lens through which to view wildlife trade, but it is the principal one in the context of regulating international wildlife trade in species that are considered to be (or to be at risk of becoming) threatened with extinction (www.cites.org). It uses a negative list approach. That is, the protection of a species (by listing on one of the three CITES appendices) requires, first, demonstration that the species meets a number of biological and trade criteria (CITES Res. Conf, 9.24 (Rev CoP17)), as a qualification for any protection under this treaty (a process that relies on accurate population data and information on ongoing trade). The negative list approach appears generous to the status quo, even for threatened species, given that monitoring can be so challenging that population size and status and the numbers traded are often unknown. Perhaps because of costs or perhaps inertia or disinterest, the required data tend to be gathered and collated only for charismatic species (e.g., elephant, rhino), although thousands of lesser-known species are largely neglected (a risk also associated with domestic trade over which CITES has no jurisdiction). Even for species that are monitored, counts are often imprecise and have limited power to detect change (e.g., Trouwborst et al. 2020).

The risks of misjudgment increase the likelihood that interventions, in the form of protective legislation under CITES—or, indeed, any other similar legislation—will arrive too late, given the numbers of species that are not yet listed (Auliya et al. 2016) or even described (see, e.g., the collection of endemic cave-dwelling invertebrates in the karst landscapes of eastern Europe; Simičevié 2017, Esmail et al. 2020).

In short, if the burden of proof lies primarily on conservationists to demonstrate that trade is negatively affecting a given species before it is afforded the legal protection required to prevent its extinction, and given the general inadequacy of monitoring, drastic overexploitation may occur long before any checks can be effective (Frank and Wilcove 2019). We have used CITES as an example, but the point applies far more widely, including to domestic wildlife laws, and to international agreements addressing other aspects affected by wildlife trade such as human health, animal welfare, or invasion risk. At a time of unprecedented human population impact, technological advancement and associated globalization, this situation calls for the application of a positive list, whereby for a species to be listed as saleable the trader must first demonstrate that trade is not detrimental to its conservation. Starting from a presumption of wildlife protection, the onus might more appropriately be put on would-be commoditisers to provide the first evidence of the trade's sustainability, humaneness, and safety, not vice versa.

## Conclusions

Nature's future will be greatly affected by the degree to which it is valued as an asset to human enterprises. Assets are valued, and things with value are, one way or another, traded. This interweaving of value and trade ensures that wildlife conservation is currently threaded through with issues of wildlife trade, for better or for worse. Currently, however, against the notably anthropocentrist definition of conservation provided by the IUCN (“Conservation is the management of human use of the biosphere so that it may yield the greatest sustainable benefit to present generations while maintaining its potential to meet the needs and aspirations of future generations. Thus, conservation is positive, embracing preservation, maintenance, sustainable use, restoration, and enhancement of the natural environment.”), and evidenced by recent reports on the state of global biodiversity (e.g., IPBES 2019), conservation is failing. Largely on the basis of our own experiences in this field, we highlighted 10 issues that we see as problematic for wildlife trade. Illegal wildlife trade is a driver of global biodiversity loss, but so too, in many cases, is legal wild animal trade, which is also diverse and huge. Legality currently guarantees neither adequate species conservation nor animal welfare standards (nor, indeed, biosecurity); relevant legislation contains loopholes, wildlife may suffer from enforcement actions, consumer demand may drive prohibited trades onto (unprotected) substitute species. Meeting demand through wildlife farming can have animal welfare consequences and enable laundering of wild-caught animals into legal markets. Demand reduction strategies do not yet work at sufficient scale, and mechanisms through which wildlife can pay for its own existence require regulations to be enforced, which they seldom are. Given humanity's increasing demands on the natural world we raise the question of how widely wildlife trade will provide solutions for conservation and suggest that wildlife trade as it currently stands is not sustainable for wildlife or for the livelihoods that it supports. In seeking a way forward, we consider the diversity of facilitators involved in wildlife trade and suggest that regulation will be needed (for those potentially beneficial forms of wildlife use such as nonconsumptive tourism), that clear responsibilities will need to be defined and those responsibilities adopted more widely. We also ask, given the burden of evidence and the urgency of the situation, whether the onus should be on traders to demonstrate that wildlife use is sustainable, and humane, rather than on conservationists to demonstrate it is not.

In raising this reverse-listing proposition, we are mindful that such a change will necessitate attending not only to feasibility (against a prevailing view, in the case of CITES, of overrestrictiveness, e.g., Abensperg-Traun 2009) but especially to unintended consequences (avoiding, e.g., the increase in rhino poaching that continues despite a ban on the trade of rhino horn; Biggs et al. 2013). Additional burdens will likely include an increase in bureaucracy, and the consequences of a multitude of bans that would come to apply to species currently traded (e.g., possibly disadvantaging people in low-income countries without the political power to influence listings). This type of dramatic managerial shift will require proactive engagement with all stakeholders at all levels of the trade chain, consideration of who can, and should, pay (which could be at the demand end of the trade chain rather than the supply end), and ensuring alternative livelihoods for those affected by changing regulations (cf. Cooney et al. 2015, Biggs et al. 2017), as well as short- and long-term land use implications (see, e.g., Child et al. 2012, Booth et al. 2021). There are examples of successful implementation of alternative livelihoods (e.g., D'Cruze et al. 2011, van Vliet 2011, although as for demand reduction interventions there is often a lack of monitoring of outcomes and impacts of these, Wicander and Coad 2018, and other local community engagement initiatives; Roe and Booker 2019). It is also important to bear in mind that where livelihoods are dependent on declining species, regardless of difficult questions about who should decide how wildlife is managed or of the appropriateness of imposing differing world views on others (cf. Vucetich et al. 2018), all of which require careful consideration; the livelihoods themselves are unsustainable (given a disappearing resource) or, in some cases, a threat to human well-being. We therefore argue that change is beneficial to people as much as to wildlife.

Given the issues raised in the present article, the growing human population and pressures on the world, this positive list may be relatively short. The point of this essay, therefore, is as much about the need for global behavior change as it is about regulating wildlife trade. Specifically, it is about the need for demand redirection (Moorhouse et al. 2020), not necessarily a reduction in use per se; a shift away from uses that are inimical to biodiversity or otherwise unethical, to those that are beneficial (including, e.g., sustainable synthetic or herbal alternatives for wild animal based traditional medicines, well regulated, noninvasive tourism practices, and others yet to be defined).

In some of the issues we raise, there are also opportunities. For example, although social media facilitates wildlife trade (e.g., Hinsley et al. 2016, Martin et al. 2018), and may play a role in driving it (Kitson and Nekaris 2017, Harrington et al. 2019), it also offers a mechanism for studying, and quantifying trade, revealing the species, export and import countries, trade routes, and network structure involved (Patel et al. 2015, Yu and Jia 2015, Hinsley et al. 2016, Martin et al. 2018, Thong et al. 2019, Marshall et al. 2020, Stringham et al. 2021), allowing targeting and prioritization of interventions and, potentially, a communication medium to inform and influence wildlife product consumers (cf. Doughty et al. 2020).

In conclusion, our purpose in this article is to stimulate the conservation community to think critically regarding global management of the trade in wildlife. In the spirit of critical friends and of collective enterprise, we encourage debate around these issues in conservation and animal welfare, and their required solutions. We note that, in this article and in raising several diverse and complex issues, each with an extensive scientific literature, the requirement for brevity inevitably causes the very simplification that we urge against. For example, we several times used CITES as a lens through which to view trade. Although CITES is a principal regulatory framework, others exist (including governmental and international enforcers of property rights and bodies such as the Marine Stewardship Council and the Forest Stewardship Council that set out production standards). Many issues are beyond the remit of CITES (which is clearly restricted to international trade in endangered species) and CITES certainly cannot be blamed for inadequacies beyond its terms of reference. Insofar as such inadequacies exist, however, they prompt the questions of whether other entities are needed to regulate them or whether the remit of CITES should be broadened. We focus largely on terrestrial wild animals (because that is where our expertise lies) but note that there are even bigger issues in the oceans (e.g., White and Costello 2014). Relevant to all systems, our overarching position is that current management approaches, and their implementation, are insufficient to guarantee sustainable, safe, and humane use of wildlife.

If sustainable wildlife commodification is the correct approach to managing human use of wildlife, it must change (see e.g. Bennet et al. 2021)—and urgently—to work for both people and wildlife. And although we have only briefly touched on the issues of disease, biosecurity, and human health, at the time of writing we come to the end of a year that has seen the most extensive global pandemic in recent history (linked, in this case, with wildlife sold at a Chinese market; Zhou et al. 2020). As a result, the world might be ready for such a change, if the correct lesson is learnt (Moorhouse et al. 2021). The journey down the road to consensus and action will not be an easy one. Indeed, insofar as protecting nature necessitates redirecting demand away from outcomes that are detrimental to wildlife, and also requires attention to the highest standards of evidence and ethics, we characterize it as demanding conservation: demanding (desiring) the best of sustainable use, a task for conservation that is demanding (difficult) but nevertheless necessary if people are to live within planetary boundaries, to coexist with wildlife, and to engage respectfully with nature.

## Author Biographical

David W. Macdonald (david.macdonald@zoo.ox.ac.uk) is the founder and director, Lauren A. Harrington is a senior researcher, Tom P. Moorhouse is a senior researcher, and Neil D'Cruze is an academic visitor at the Wildlife Conservation Research Unit, in the Department of Zoology at the University of Oxford, in Tubney, Oxfordshire, United Kingdom. Neil D'Cruze is also the global head of wildlife research at World Animal Protection, in London, England.
